# The effect of solid fuel use on childhood mortality in Nigeria: evidence from the 2013 cross-sectional household survey

**DOI:** 10.1186/1476-069X-13-113

**Published:** 2014-12-16

**Authors:** Osita Kingsley Ezeh, Kingsley Emwinyore Agho, Michael John Dibley, John Joseph Hall, Andrew Nicolas Page

**Affiliations:** School of Medicine, University of Western Sydney, Locked Bag 1797, Penrith, NSW 2571 Australia; School of Science and Health, University of Western Sydney, Locked Bag 1797, Penrith, NSW 2571 Australia; Sydney School of Public Health, University of Sydney, Edward Ford Building (A27), Sydney, NSW 2006 Australia; School of Medicine and Public Health, Faculty of Health, University of Newcastle, Callaghan, NSW 2308 Australia

**Keywords:** Indoor air pollution, Solid fuels, Nigeria, Childhood mortality

## Abstract

**Background:**

In Nigeria, approximately 69% of households use solid fuels as their primary source of domestic energy for cooking. These fuels produce high levels of indoor air pollution. This study aimed to determine whether Nigerian children residing in households using solid fuels at <5 years of age were at higher risk of death.

**Methods:**

The 2013 Nigeria Demographic and Health Survey data were analysed in Cox regression analyses to examine the effects of solid fuel use on deaths of children aged 0–28 days (neonatal), 1–11 months (post-neonatal), and 12–59 months (child).

**Results:**

The results indicated that approximately 0.8% of neonatal deaths, 42.9% of post-neonatal deaths, and 36.3% of child deaths could be attributed to use of solid fuels. The multivariable analyses found that use of solid fuel was associated with post-neonatal mortality (hazard ratio [HR] =1.92, 95% confidence interval [CI]: 1.42–2.58) and child mortality (HR = 1.63, CI: 1.09–2.42), but was not associated with neonatal mortality (HR = 1.01, CI: 0.73–1.26). Living in rural areas and poor households were associated with an increased risk of death during the three mortality periods.

**Conclusion:**

Living in a rural area and poor households were strongly associated with an increased risk of a child > 1 to < 60 months dying due to use of solid fuels. The health effects of household use of solid fuels are a major public health threat that requires increased research and policy development efforts. Research should focus on populations in rural areas and low socioeconomic households so that child survival in Nigeria can be improved.

## Background

Indoor air pollution (IAP) emanating from burning solid fuels (wood, charcoal, animal dung, coal and crop waste) for cooking and home heating remains a major environmental and public health challenge in developing countries. Worldwide, approximately 4.3 million people have died as a result of illnesses attributed to IAP; these deaths include 534,000 children <5 years of age [1]. Most of the deaths occur in low- and middle-income countries, including Nigeria. However, the use of solid fuels and health effects of the mix of chemical components (e.g., carbon monoxide, sulphur oxides, nitrogen oxides, particulates, benzene, formaldehyde, polyaromatic compounds, arsenic, lead) from solid fuel use [2, 3] are not well understood even though household indoor cooking could result in high levels of these chemical components. Children <5 years of age are one of the vulnerable groups most likely to experience ill health caused by solid fuel use, as they are with their mothers whilst they are cooking [4].

Results of previous studies have indicated that children <5 years of age living in homes using solid fuels for cooking are at a greater risk of dying from acute respiratory illnesses [5, 6]. Respiratory and immune system development occurs during this period, which may impair the immune response and lead to more severe infection and progression into the lower respiratory, particularly during the first year of life [7, 8]. In 2014, the World Health Organization (WHO) indicated that >50% of the deaths of children <5 years of age were attributed to acute lower respiratory infections triggered by solid fuels use [1]. Poverty is one of the main drivers for solid fuel use in many low- and middle-income countries.

Evidence from the Nigeria Demographic and Health Surveys (NDHS) indicated that in the past decade (from 2003–2013), the proportion of Nigerian households using solid fuels as their primary source of domestic energy for cooking remained constant at approximately 69% [9, 10]. Each year, over 95,000 Nigerians, including children <5 years of age, die from exposure to firewood smoke [11]. The health effects of household solid fuel use, and the increased risk of childhood mortality, have been somewhat neglected by public health researchers and policy makers in Nigeria.

We present new and emerging evidence on the effects of solid fuel use on childhood mortality in Nigeria. We examined the relationship between household solid fuel use and death rate of children <5 years for age groups (0–28 days, 1–11 months, and 12–59 months) because the effects of environmental, demographic, and socioeconomic factors may be amongst these groups [12, 13].

## Methods

The dataset used for this study was from the 2013 NDHS. The dataset is available online from ICF international, Rockville, MD, USA, after ethics approval is obtained [10]. The National Population Commission (NPC) conducts the NDHS in conjunction with ICF International. The United States Agency for International Development (USAID) is the main sponsor of the NDHS. Approximately every 5 years, the NDHS collects information on demographic and health issues (e.g., maternal and child health, childhood mortality, and education) from nationally representative households located in rural and urban areas. The 2013 NDHS data were obtained by interviewing eligible reproductive age women and men, aged 15–49 and 15–59 years, respectively, who participated in the household survey. The information obtained was recorded in three questionnaires (i.e., household, and women’s and men’s questionnaires). The detailed sampling procedures used in the NDHS have previously been published [10].

A total of 38,522 households were interviewed during the 2013 NDHS survey period. Of these households, 38,948 women and 17,359 men were successfully interviewed, corresponding to response rates of 97.6% and 95.2%, respectively. A total of 30,726 singleton live-births were reported for the 5-year period prior to the interview date. Multiple births were excluded from our analysis because compared with singletons, multiple births are associated with higher childhood mortality risk [14, 15]. The analysis was restricted to births that occurred within the previous 5 years to minimise recall bias in the birth and death dates reported by the mothers. In addition, children < 5 years of age are most likely to be indoors under their mother’s supervision whilst cooking occurs.

### Descriptive study variables

#### Outcome variables

The main outcome variables were neonatal mortality (death between birth and 28 days of age), post-neonatal mortality (death between 1 and 11 months of age) and child mortality (death between 12 and 59 months of age). Binary outcome variables were used in the analysis. Child death was considered a ‘case’ (= 1) if death occurred during the specified age period and a ‘non-cases’ (= 0) if the child was alive throughout the specified age period.

### Main study factor

The main study factor was the main type of cooking fuels available to household members at the time of survey. The respondents were asked “What type of fuel does your household mainly use for cooking”, which was followed by 11 categories of cooking fuels, including electricity, liquefied petroleum gas, natural gas, biogas, kerosene, coal/lignite, charcoal, wood, straw/shrubs/grass, agricultural crop and animal dung. For the analysis, these 11 categories were classified into two groups based on NDHS definitions, solid (coal/lignite, charcoal, wood, straw/shrubs/grass, agricultural crop, animal dung) and non-solid (electricity, liquefied petroleum gas (LPG), natural gas, biogas, kerosene) fuels.

### Potential confounding variables

The potential confounding variables considered were based on previous literature on the effects of cooking fuels on childhood mortality [16–18], particularly in developing countries. These variables were adapted to the data available in the 2013 NDHS. Potential child-related confounders were sex, mother’s perception of her newborn’s size at birth, and breastfeeding. The child’s sex was also included in the analysis because results of a study in Bangladesh indicated that boys spend less time indoors compared with girls [19]. Therefore, girls are more likely to inhale IAP than boys, and girls are more likely than boys to be admitted for acute respiratory diseases [20]. The mother’s perception of her newborn’s size was categorised into two groups (small or very small, and average or large), and was used as a proxy for birth weight because >50% of children are not weighed at birth [10]. Results of recent studies in India and Guatemala indicated that children born in households using high-polluting solid fuels were 73 g and 63 g, respectively, lower in birth weight compared with children born in households using low-polluting fuels [21–23]. We considered breastfeeding in the analysis because it has a protective effect on the risk of respiratory disease [24, 25], especially in the first year of an infant’s life.

Confounders associated with maternal characteristics included education, working status and age at the birth of the child. Level of the mother’s education is correlated with child survival, particularly during the post-neonatal and child period [26, 27]. Compared with less educated mothers, educated mothers were more likely to obtain paid employment, which results in household members being exposed to solid and non-solid fuel combustion for shorter periods of time. Educated mothers can understand the health hazards of solid fuel use, and are more likely to purchase non-solid fuels. Educated mothers are also more likely to appropriately use healthcare services than their less educated peers. The level of education attained by mothers was categorised into three groups (no education, primary and secondary or higher education). Employment status was divided into two categories (working and not working). Mother’s age at birth of the child was adjusted for because the frequency of exposure to cooking fuel smoke may vary as mothers grow older. It was categorised into four groups (age <20, 20–29, 30–39 and 40–49 years).

The respondent’s living location at the time of the survey was used to classify the residence, and was classified as urban or rural. More than two-thirds of households using solid fuels are in rural areas [10, 28]. Household economic status is associated with use of high-polluting fuels [29]. As higher income levels are achieved, households are more likely to switch to more modern stoves and cleaner fuels, regardless of cultural traditions [30]. A household wealth index variable was constructed using household assets, which were weighted using a principal component analysis [31]. The assets considered were presence of a television, radio, refrigerator, telephone, car, bicycle, motorcycle and canoe, and ownership of agricultural land, a livestock farm or a bank account. In the NDHS data set, the household wealth variable was categorised into five quintiles: poorest, poorer, middle, richer, and richest. However, in the analysis the household wealth index was re-categorised into three groups. The bottom 40% of households was arbitrarily referred to as poor households, the next 40% as middle-income households, and the top 20% as high-income households.

Location of household kitchen was categorised into three groups (separate building, outdoor and in the house). However, frequency of cooking, ventilation facility, and duration of cooking variables were not included in this analysis because they were not collected. The previously mentioned potential confounding variables were used to measure the effects of use of solid fuels on neonatal, post-neonatal and child mortality.

### Statistical analysis

Initially, mortality rates categorised by the two groups based on type of cooking fuel were estimated using a method similar to that described by Rutstien and Rojas [32]. Cox proportional hazards regression models were then used for multivariable analyses that independently examined the effect of each factor after adjusting for potential confounding variables.

A staged modelling technique was employed in the multivariable modelling. In the first stage, all potential confounding variables were entered into the baseline multivariable model to assess their relationship with the study outcomes. A stepwise backwards elimination process was performed, and variables that were associated with the study outcomes at a 5% significance level were retained in the model (model 1). In the final stage of the analysis, the main study factor (type of cooking fuels) was entered into model 1, and variables with a p-value <0.05 were retained in the final model (model 2).

The hazard ratios (HRs) and their 95% confidence intervals (CIs) obtained from the adjusted Cox proportional hazards models were used to measure the effect of the type of cooking fuels on neonatal, post-neonatal and child mortality. The “SVY” commands in STATA version 12.0 (Stata Corporation, College Station, TX, USA) were used in all analyses to adjust for the cluster sampling survey design, weights, and the calculation of standard errors.

The population attributable risk (PAR) was calculated to estimate total risk of neonatal, post-neonatal and child mortality in the general population that was attributable to household air pollution from smoke emanating from solid fuel use between 2009 and 2013. PAR was obtained using the following formula, which is recommended for multivariate-adjusted relative risks [33, 34]:


where aHR was the adjusted hazard ratio for (neonatal, post-neonatal, and child) mortality from use of solid fuel.

## Results

A weighted total of 30,726 singleton live-births of children occurred within the 5-year period prior to the 2013 NDHS survey interview date. Of the total live births, 2,615 children died within the 5-year period. These deaths consisted of 1,011 neonates (first day of life to 28 days of age), 789 post-neonates (between 1 and 11 months of age), and 815 young children (between 12 and 59 months of age).

Table 1 presents the results for the percentage distribution of neonatal, post-neonatal, and young child deaths by selected background characteristics. In the 2013 NDHS, approximately 82% of neonates died in households using solid fuels for cooking. This number increased to 90% for the post-neonatal group and was 94% for the young child group. Wealthy households had the lowest percent of deaths compared with poor and middle-income households (13.8% neonatal, 9.0% post-neonatal, and 4.2% child). Greater than 70% of the neonatal, post-neonatal, and child deaths occurred in the rural areas.

**Table 1:** **Percentage distribution of neonatal, post-neonatal and child mortality by background characteristics**

Variables	Neonatal deaths (n = 1,011)	Post-neonatal deaths (n = 789)	Child deaths (n = 815)
**Residence type**			
Urban	29.1	26.9	17.1
Rural	70.9	73.1	82.9
**Household wealth index**			
Poor	52.2	60.6	71.2
Middle	34.0	30.4	24.6
Rich	13.8	9.0	4.2
**Mother's education**			
No education	52.1	59.8	69.2
Primary	21.9	17.5	17.5
Secondary or higher	25.9	22.7	13.3
Mother's working status*			
Not working	35.0	33.9	33.2
Working	64.5	66.1	66.8
**Mother's age**			
< 20	7.9	7.1	4.8
20―29	47.4	44.8	46.1
30―39	33.3	36.3	36.7
40―49	11.4	11.8	12.4
Mother's perceived baby size*			
Small or very small	23.5	16.9	18.7
Average or larger	68.2	76.9	76.0
**Sex**			
Female	42.6	49.0	48.7
Male	57.4	51.0	51.3
**Currently breastfeeding**			
Yes	28.7	35.6	43.1
No	71.3	64.4	56.9
Location of Kitchen*			
Separate building	17.6	19.7	16.1
Outdoor	22.8	25.2	22.6
House	59.3	54.3	61.2
Type of cooking fuel*			
Solid fuel	82.2	89.6	93.8
Non-solid fuel	16.4	9.6	5.6

*Percentages did not add up to 100% because of missing values.

The neonatal mortality rate (NMR) was higher among neonates born to mothers in households using solid fuels for cooking (NMR: 33.4 vs 29.6). The post-neonatal mortality rate (PMR) in households using solid fuels for cooking was greater than in households using non-solid fuels (PMR: 28.4 vs 13.6). Similarly, the child mortality rate (CMR) for children aged 12–59 months was higher in households using solid fuels for cooking compared with households not using solid fuels for cooking (CMR: 30.7 vs 8.2).

Approximately, 0.8% of neonatal deaths (PAR: 0.8; CI: −7.8―2.8), 43% of post-neonatal deaths (PAR: 42.9; CI: 31.9―61.4) and 36% of child deaths (PAR: 36.3; CI: 33.1―52.1) for the 5-year period prior to the 2013 NDHS survey may be attributed to the use of solid fuels.

### The effect of solid fuel use on neonatal mortality

Table 2 presents the results for the effect of cooking fuel on neonates survival after adjusting for potential confounding factors. Neonates born to mothers in households using solid fuels for cooking had a slightly higher risk of neonatal death (HR = 1.01; CI: 0.73―1.26) compared with neonates in households using non-solid fuel. This difference was not statistically significant.

**Table 2:** **Neonatal mortality model**

Variables	(Model 0)^n^	(Model 1)‴	(Model 2)^^~^
	HR (95% CI)	HR (95% CI)	HR (95% CI)
**Residence type**			
Urban	Ref	Ref	
Rural	1.36(1.13―1.65)	1.30(1.07―1.58)	1.32(1.06―1.64)
**Household wealth index**			
Rich	Ref		
Poor	1.43(1.09―1.88)		
Middle	1.14(0.86―1.52)		
**Mother's education**			
Secondary or higher	Ref		
No education	1.26(1.01―1.56)		
Primary	1.20(0.94―1.52)		
**Mother's working status**			
Not working	Ref		
Working	0.76(0.64―0.91)		
**Mother's age**			
40―49	Ref	Ref	
< 20	3.14(2.09―4.70)	3.17(2.12―4.74)	3.16(2.12―4.74)
20―29	1.11(0.81―1.51)	1.22(0.90―1.66)	1.22 0.90―1.65)
30―39	0.90(0.64―1.24)	0.99(0.71―1.37)	0.98(0.71―1.36)
**Mother's perceived baby size**			
Average or larger	Ref	Ref	Ref
Small or very small	1.95(1.63―2.34)	1.86(1.55―2.24)	1.86(1.55―2.24)
**Sex**			
Female	Ref	Ref	Ref
Male	1.31(1.11―1.55)	1.33(1.13―1.57)	1.33(1.13―1.64)
**Breastfeeding currently**			
Yes	Ref	Ref	Ref
No	1.98(1.64―2.38)	2.12(1.76―2.55)	2.12(1.75―2.55)
**Location of kitchen**			
Separate building	Ref	Ref	Ref
Outdoors	0.88(0.68―1.15)		
House	1.15(0.92―1.44)		
**Cooking fuel**			
Non-Solid fuel	Ref		Ref
Solid fuel	1.16(0.91―1.47)		1.01(0.73―1.26)

^^^Independent variables adjusted were: place of residence, wealth index, child size, child’s gender, currently. Breastfeeding and mother’s (education, working status, age); ^n^Model 0 – unadjusted independent variables; ‴Model 1 – independent variables associated with neonatal mortality; Model 2 – Model 1 plus type of cooking fuels; ^**~**^Missing values were excluded from model 0, 1, and 2.

Table 2 (model 2) presents the results for other significant factors that affected neonatal deaths. These factors were neonates born to mothers <20 years of age (HR = 3.16; CI: 2.12–4.74), neonates whose body size was perceived by the mother as small or smaller (HR = 1.86; CI: 1.55–2.24), male neonates (HR = 1.33; CI: 1.13–1.64), neonates born to mothers residing in rural areas (HR = 1.32; CI: 1.06–1.64), and neonates not currently breastfed (HR = 2.12; CI: 1.75–2.55). When the place of residence was replaced by the household wealth index in the final model, children from poor households had a significantly high risk of infant death (HR = 1.55; CI: 1.13–2.13).

### The effect of solid fuel use on post-neonatal mortality

Table 3 presents the results for the effect of cooking fuel on post-neonatal mortality after adjustment for confounding factors. Compared with the reference category (Table 3, model 2), infants in households cooking with solid fuels (HR = 1.92; CI: 1.42–2.58) had a significantly higher risk of post-neonatal mortality.

**Table 3:** **Post-neonatal mortality model**

Variables	(Model 0)^n^	(Model 1)‴	(Model 2)^^~^
	HR (95% CI)	HR (95% CI)	HR (95% CI)
**Residence type**			
Urban	Ref	Ref	Ref
Rural	1.48 (1.17―1.88)	1.44(1.15―1.80)	1.16(1.01―1.46)
**Household wealth index**			
Rich	Ref		
Poor	2.48 (1.78―3.45)		
Middle	1.63 (1.16―2.29)		
**Mother's education**			
Secondary or higher	Ref		
No education	1.51 (1.20―1.91)		
Primary	1.13 (0.85―1.50)		
**Mother's working status**			
Not working	Ref		
Working	0.79 (0.67―0.94)		
**Mother's age**			
40―49	Ref	Ref	
< 20	3.76 (2.45―5.79)	3.62(2.41―5.45)	3.63(2.41―5.46)
20―29	1.12 (0.84―1.52)	1.17(0.87―1.57)	1.19(0.89―1.60)
30―39	0.99 (0.73―1.34)	1.03(0.77―1.39)	1.07(0.80―1.45)
**Mother's perceived baby size**			
Average or larger	Ref		
Small or very small	1.28 (1.04―1.59)		
**Sex**			
Female	Ref		
Male	0.98 (0.82―1.18)		
**Breastfeeding currently**			
Yes	Ref	Ref	Ref
No	1.38 (1.14―1.67)	1.41(1.18―1.69)	1.46(1.22―1.74)
**Location of kitchen**			
Separate building	Ref		
Outdoors	0.92 (0.69―1.22)		
House	0.90 (0.71―1.14)		
**Cooking fuel**			
Non-solid fuel	Ref		Ref
Solid fuel	2.16 (1.58―2.96)		1.92(1.42―2.58)

^^^Independent variables adjusted were: place of residence, wealth index, child size, child’s gender, currently breastfeeding and mother’s (education, working status, age); ^n^Model 0 – unadjusted independent variables; ‴Model 1 – independent variables associated with post-neonatal mortality; Model 2 – Model 1 plus type of cooking fuels; ^**~**^Missing values were excluded from model 0, 1 and 2.

Model 2 (Table 3) revealed significant factors, other than households using solid fuels for cooking, that affected post-neonatal deaths. There was a significantly higher risk of infant death for infants born to mothers residing in rural areas (HR = 1.16; CI: 1.01–1.46), compared with infants in urban areas. When the place of residence was replaced by a household wealth index in the final model, infants born to mothers from poor households had a significantly high risk of infant death (HR = 1.81; CI: 1.15–2.83). Infants born to mothers <20 years of age had a 3.63 times greater risk of dying compared with infants of mothers aged ≥20 years (HR = 3.63; CI: 2.41–5.46). There was a significantly higher risk of post-neonatal death for infants not currently breastfed (HR = 1.46; CI: 1.22–1.74).

### The effect of solid fuel use on child mortality

Table 4 presents the results for the effect of cooking fuels on young children after adjustment for confounding factors. As indicated in model 2 results, children aged between 12 and 59 months and living in households using solid fuels for cooking had a greater risk of child mortality (HR = 1.63; CI: 1.09–2.42), compared with children in households using non-solid fuels.

**Table 4:** **Child mortality model**

Variables	(Model 0)^n^	(Model 1)‴	(Model 2)^^~^
	HR (95% CI)	HR (95% CI)	HR (95% CI)
**Residence type**			
Urban	Ref	Ref	Ref
Rural	2.45(1.95―3.07)	1.75(1.39―2.22)	1.59(1.25―2.03)
**Household wealth index**			
Rich	Ref		
Poor	4.80(3.89―8.64)		
Middle	2.52(1.64―3.88)		
**Mother's education**			
Secondary or higher	Ref	Ref	Ref
No education	3.20(2.45―4.19)	2.46(1.85―3.26)	2.13(1.58―2.87)
Primary	1.99(1.46―2.73)	1.74(1.26―2.39)	1.55(1.12―2.15)
**Mother's working status**			
Not working	Ref		
Working	0.94(0.77―1.14)		
**Mother's age**			
40―49	Ref		
< 20	1.60(0.99―2.58)		
20―29	0.92(0.70―1.19)		
30―39	0.88(0.67―1.16)		
**Mother's perceived baby size**			
Average or larger	Ref	Ref	
Small or very small	1.45(1.15―1.82)	1.26(1.01―1.58)	
**Sex**			
Female	Ref		
Male	1.06(0.89―1.26)		
**Breastfeeding currently**			
Yes	Ref		
No	0.97(0.83―1.15)		
**Location of kitchen**			
Separate building	Ref		
Outdoors	1.16(0.86―1.57)		
House	1.40(1.08―1.81)		
**Cooking fuel**			
Non-solid fuel	Ref		Ref
Solid fuel	3.48(2.46―4.93)		1.63(1.09―2.42)

^^^Independent variables adjusted were: place of residence, wealth index, child size, child’s gender, currently breastfeeding and mother’s (education, working status, age); ^n^Model 0 – unadjusted independent variables; ‴Model 1 – independent variables associated with post-neonatal mortality; Model 2 – Model 1 plus type of cooking fuels; ^**~**^Missing values were excluded from model 0, 1, and 2.

There were other factors associated with a significantly higher risk of child mortality (Table 4, model 2). Children had a significantly higher risk of death if their mothers had no formal education (HR = 2.13; CI: 1.58–2.87) or had a primary education (HR = 1.55; CI: 1.12–2.15). A significantly higher risk of death was also observed for children whose mothers resided in rural areas (HR = 1.59; CI: 1.25–2.03). When the place of residence was replaced by the household wealth index in the final model, children from poor households had a significantly high risk of child death (HR = 3.73; CI: 2.07–6.73).

## Discussion

Household use of solid fuels was associated with an increased risk of neonatal, post-neonatal and child mortality after controlling for potential confounders including household wealth status, place of residence, mother’s level of education, mother’s perceived size of her child at birth, sex, breastfeeding, mother’s age and mother’s employment status. Household use of solid fuels was not significantly associated with neonatal mortality. These findings are consistent with the results of a study conducted in India, which indicated that the association between household use of solid fuels and neonatal mortality is not significant [35]. Explanations for the lack of association include contribution from biological factors, such as low birth weight and prematurity, and complications related to pregnancy and delivery [36], rather than household environmental health hazards. Breastfeeding, including exclusive breastfeeding, may also protect against the development of respiratory diseases [25, 37]. The strong effect of breastfeeding observed during the neonatal and post-neonatal mortality periods examined in this study reaffirms the protective effects of breastfeeding on improving the growth, health, and survival of children <5 years of age.

The effect of household use of solid fuels on mortality increased significantly during the post-neonatal period. This may be attributable to the fact that infants in their first year of life are usually carried on their mother’s back or stand beside their mother while she is cooking, thus exposing the infant to high concentrations of pollutants from solid fuel for considerable periods. This result is in line with observations from a case–control study performed in Gambia that found that there is a significantly higher risk of mortality from acute lower respiratory infection among children often carried on their mother’s back during cooking [38]. This practice is common in sub-Saharan African countries, including in Nigeria. Close proximity to a pollution source and time spent in the vicinity is likely to increase the level of exposure to pollution, which may lead to adverse health problems [39]. We noted that household use of solid fuels increased the risk of death for post-neonates by 92%, compared with a 63% increased risk for children between 12 and 59 months. Compared with the infancy period, the lower mortality risk reported during the child period could be linked to their relatively well-developed lungs and immune response to pathogens [40].

Findings from this study indicate that during all age periods, children <5 years residing in rural areas had a significantly higher risk of mortality compared with their peers in urban areas. This finding is supported by results from other studies [41, 42], which indicated that residence in rural areas is a strong predictor of mortality of children <5 years of age. Clear urban–rural differences in household use of solid fuel are apparent in many sub-Saharan African countries. More than two-thirds of rural dwellers rely exclusively on gathered wood, charcoal, animal dung, crop waste, and coal waste for domestic energy [10, 28]. Socioeconomic status (e.g., poor households) is one of the major factors affecting solid fuel use [29]. The significant effect of economic status was also apparent in all three age groups when place of residence was replaced by household wealth index in the final model. This finding reaffirms that wealth has a positive effect on child survival. Limited access to cleaner energy (electricity, LPG, gas) may also hinder rural residents from using efficient cooking fuels. Only 34% of the rural residents in Nigeria have access to electricity [10].

In addition to solid fuel use, other factors that were significantly associated with an increased risk of neonatal, post-neonatal, and child mortality included the mother’s perception of her newborn’s size at birth (small or very small), sex (male neonate), maternal age (<20 years), and mother’s level of education (no education). These results are consistent with the results of previous studies that examined the effect of cooking fuels on childhood mortality [18, 23, 41, 43].

It is possible that the estimates we reported in our study may have been underestimated because of the following reasons. (1) Children aged between 1 and 4 years are more likely to move around, as a result they may be exposed to both household and ambient air pollution. (2) NDHS did not gather information about the history of cooking fuel use. This is imperative because previous study indicated that household income is a strong predictor for switching to cleaner fuels [30].

This study has some limitations. First, data on households that use a combination of solid and non-solid fuels were not available from the NDHS database, and misclassification of use of cooking fuels may have occurred. Results of a study performed in India revealed that households reporting kerosene as their primary fuel for domestic energy but that they frequently switched to solid fuels reported higher levels of exposure to household air pollution [44]. A second limitation was that causal effects could not be measured because the analyses were based on a retrospective cross-sectional study. The third limitation was that detailed health assessments of the child and mother were not available at the time of the survey. Finally, other important variables such as ventilation facility, duration of cooking, and frequency of cooking were not used in this analysis because they were not collected in the 2013 NDHS. Strengths of this study included that the indicators used for cooking fuels was based on WHO recommendations. Recall errors arising from dates of birth and death given by mothers interviewed during the survey were minimised by restricting the analyses to births within the 5-year period preceding the survey. Data used in this study were from a nationally representative survey, which had a 97.6% response rate.

## Conclusions

Our analyses examined whether children <5 years of age residing in households using solid fuels were at higher risk of death. The results indicate that use of solid fuels increased the risk of post-neonatal and child deaths.

In addition to the effect of solid fuel use on childhood mortality, children from households in rural areas, children from poor households, children delivered by younger mothers (<20 years of age), children perceived by their mothers to have been smaller than average at birth, and children with illiterate mothers had a significantly higher risk of neonatal, post-neonatal and child mortality. Findings from this study indicate the need to create public awareness of the health risks of using solid fuel and to implement community-based domestic energy interventions. To improve child survival in Nigeria, these interventions should target rural and low socioeconomic status households.

## Abbreviations

IAP: Indoor air pollution
NDHS: Nigeria demographic and health survey
NPC: National population commission
WHO: World health organisation
LPG: Liquefied petroleum gas
HR: Hazard ratio
aHR: Adjusted hazard ratio
PAR: Population attributable risk
CI: Confidence interval
NMR: Neonatal mortality rate
PMR: Post-neonatal mortality
CMR: Child mortality rate.
